# Medical and Surgical Directory of the United States

**Published:** 1886-10

**Authors:** 


MEDICAL AND SURGICAL DIRECTORY OF THE UNITED STATES. *Published by* R. L. POLK & CO, *Detroit.*

This work contains the names of all the physicians in the United States, arranged alphabetically and by localities. In addition the local and state laws relating to physicians and public health, medical colleges, medical journals, national, state and local medical societies, and, in fact, all information relating to physicians is given in a very full manner. The work is well arranged and easy of reference. It represents an almost incalculable amount of work in accumulating the data upon which it is based, and is of great value to physicians and to others. The publishers deserve a substantial return for the good work they have done in preparing and issuing this directory, which seems to be exceptionally accurate.

